# A crystallographic study of crystalline casts and pseudomorphs from the 3.5 Ga Dresser Formation, Pilbara Craton (Australia)

**DOI:** 10.1107/S1600576718007343

**Published:** 2018-07-05

**Authors:** Fermin Otálora, A. Mazurier, J. M. Garcia-Ruiz, M. J. Van Kranendonk, E. Kotopoulou, A. El Albani, C. J. Garrido

**Affiliations:** a Laboratorio de Estudios Cristalográficos, Instituto Andaluz de Ciencias de la Terra (CSIC-UGR), Avenida de las Palmeras, 4, Armilla, Granada, Spain; b IC2MP, UMR CNRS 7285, Equipe E2 HydrASA, Université de Poitiers, 5 rue Albert Turpain, Bâtiment B8, TSA 51106, 86073 Poitiers Cedex 9, France; c School of Biological, Earth and Environmental Sciences and Australian Centre for Astrobiology, University of New South Wales Sydney, Kensington, NSW 2052, Australia

**Keywords:** aragonite, pseudomorphs, crystal morphology, X-ray tomography, precambrian

## Abstract

Crystallography has a long history of providing knowledge and methods for applications in other disciplines. The identification of minerals using X-ray diffraction is one of the most important contributions of crystallography to earth sciences. However, when the crystal itself has been dissolved, replaced or deeply modified during the geological history of the rocks, diffraction information is not available. Instead, the morphology of the crystal cast provides the only crystallographic information on the original mineral phase and the environment of crystal growth. This article reports an investigation of crystal pseudomorphs and crystal casts found in a carbonate-chert facies from the 3.48 Ga-old Dresser Formation (Pilbara Craton, Australia), considered to host some of the oldest remnants of life. A combination of X-ray microtomography, energy-dispersive X-ray spectroscopy and crystallographic methods has been used to reveal the original phases of these Archean pseudomorphs. It is found with a high degree of confidence that the original crystals forming in Archean times were hollow aragonite, the high-temperature polymorphs of calcium carbonate, rather than other possible alternatives such as gypsum (CaSO_4_·2H_2_0) and nahcolite (NaHCO_3_). The methodology used is described in detail.

## Abstract 2

Crystallographic methods are used to identify the primary mineral phase of pseudomorphs of crystals embedded in 3.48 Ga bedded carbonate-chert rocks from the Dresser Formation, Pilbara Craton, Australia. This identification provides valuable information on the chemical environments at the onset of life on Earth.

## 1. Introduction   

The identification of minerals and the chemical environment in which they grew is one of the main contributions of crystallography and crystal growth to geosciences (García-Ruiz & Otálora, 2014 ▸). The difficulty of these studies scales with the age and alteration state of the rocks. Our planet is known to be 4.56 billions years old (Halliday, 2000 ▸; Dalrymple, 2001 ▸). The period elapsing from the origin of the planet to 4.0 billion years ago is called the Hadean. Minerals and rocks older than 4.03 Ga are not preserved, with the exception of a few Hadean detrital zircon crystals found within younger rocks (Amelin *et al.*, 1999 ▸; Wilde *et al.*, 2001 ▸; Valley *et al.*, 2014 ▸). During geological history, most Archean (4.0–2.5 Ga) and Proteorozoic (2.5–0.56 Ga) rocks have undergone a number of geochemical and tectonic processes (including weathering, diagenesis, metamorphism, deformation *etc*.) that have obliterated much of the information on the geological, chemical and physical conditions during the original deposition of ancient sedimentary rocks.

The mineral phases, the chemical composition and the textures of the rocks found in surface outcrops are commonly very different from those of the original sediment. Therefore, revealing the original information encoded in crystals of the oldest rocks is a formidable task, but worth pursuing, particularly when dealing with rocks that contain the oldest remnants of life on Earth. The detection of primitive life, in the form of either microfossils, carbonaceous structures or induced carbonate structures like the stromatolites, is by itself a challenging subject, requiring different lines of investigation. Providing accurate and precise information about the environment in which these putative fossils formed is key for a correct identification of the primitive organisms and the ecosystems in which they flourished.

**Figure 1:**
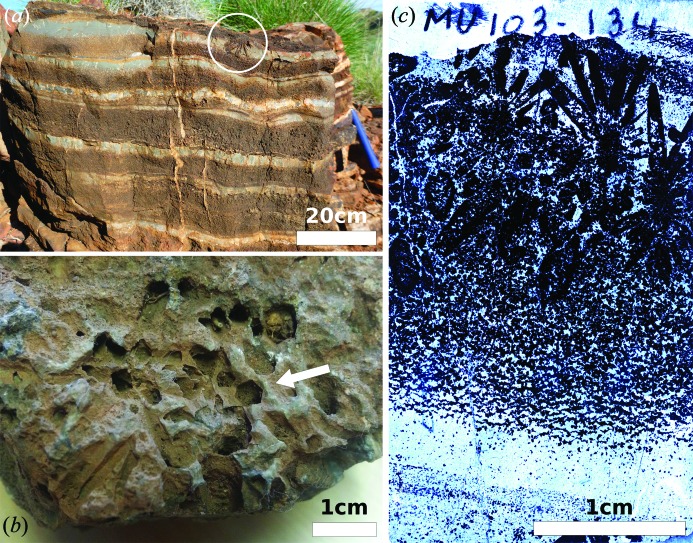
(*a*) *In situ* outcrop of the bedded carbonate-chert ‘zebra rock’ in the Dresser Formation (Locality: UTM Zone 50 K E753868, N7665540). This view, perpendicular to bedding, shows the alternating carbonate (brown)–chert (white) bedding and the presence of radiating crystal splays (circle). (*b*) Detail of crystal pseudomorphs in a hand sample, looking at a plane close to bedding. Hollow casts of hexagonal or pseudohexagonal pseudomorphic crystals forming radial crystal aggregates are clearly visible (white arrow). (*c*) Thin section of the sample (transmitted light microscopy), showing the hollow crystal splays and the lamination.

The *ca* 3.48 Ga Dresser Formation and associated network of chert-barite hydro­thermal veins in the North Pole Dome region of the East Pilbara Terrane, Pilbara Craton (Van Kranendonk *et al.*, 2002 ▸; Van Kranendonk *et al.*, 2007 ▸), are well known for containing Earth’s most convincing, oldest evidence of life in the form of morphologically variable stromatolites (Walter *et al.*, 1980 ▸; Van Kranendonk *et al.*, 2007 ▸, 2008 ▸; Van Kranendonk, 2011 ▸), putative microfossils and microbial mats (Ueno *et al.*, 2001 ▸, 2004 ▸; Glikson *et al.*, 2008 ▸), and fractionated stable isotopes (Ueno *et al.*, 2004 ▸, 2006 ▸, 2008 ▸; Shen *et al.*, 2001 ▸, 2009 ▸; Philippot *et al.*, 2007 ▸). The Dresser Formation contains also large (mm to cm), radiating crystal pseudomorphs within a unit of interbedded carbonate and chert (Fig. 1 ▸ *a*) (Buick & Dunlop, 1990 ▸; Van Kranendonk *et al.*, 2008 ▸). Additional macroscopic idiomorphic crystals have been described in the ‘bedded barite lithofacies’ (sub vertical barite crystals, 2–10 mm wide, 10–250 mm long, arranged as palisades of parallel crystals or in adjoining fans of subvertical crystals), the ‘gypsiferous lutite lithofacies’ (0.1–5.0 mm wide crystals scattered throughout silty mudstone) and the ‘gypsiferous peloidal chert’ lithofacies (rosettes of radiating crystal pseudo­morphs up to 20 mm in diameter with individual crystals having a core of incorporated sedimentary material).

The identification of these pseudomorphic crystals has been rather controversial. Several authors (Groves *et al.*, 1981 ▸; Buick & Dunlop, 1990 ▸; Lambert *et al.*, 1978 ▸) interpreted the crystal rosettes of the Dresser Formation as gypsum crystals formed by the evaporation of the seawater in a shallow marine basin. However, the same crystals have been alternatively interpreted as magnesite (Bone, 1983 ▸) or aragonite (Grotzinger, 1989 ▸). Runnegar *et al.* (2001 ▸) showed that barite crystals were primary, and not replacive of gypsum. Van Kranendonk *et al.* (2008 ▸) identified aragonite within the cores of the crystal splays from the ‘zebra rock’ unit, using optical light microscopy, but noted that this looked to be a replacement of an earlier mineral phase. Similar (pseudo-)hexagonal crystals are also known from elsewhere in Pilbara, where they have been interpreted as sodium bicarbonate (nahcolite), precipitated under evaporative settings (Sugitani *et al.*, 2003 ▸). These controversies highlight the importance of a conclusive identification of the primary mineral phase and an understanding of the different methodologies used to reach the conclusion. In the case of the Dresser Formation crystal splays, their correct identification – whether grown during deposition of the sediments and/or during early diagenesis – is important for establishing the composition of the water body and the ocean/atmosphere environment at this early period of Earth history.

In this work, we present the results of a direct characterization of the pseudomorph crystals and crystal casts found in the Dresser Formation chert-carbonate rocks, and also discus the methodological issues involved in the characterization and identification of crystal pseudomorphs embedded in very old rocks.

## 2. Mineralogical and petrological characterization   

**Figure 2:**
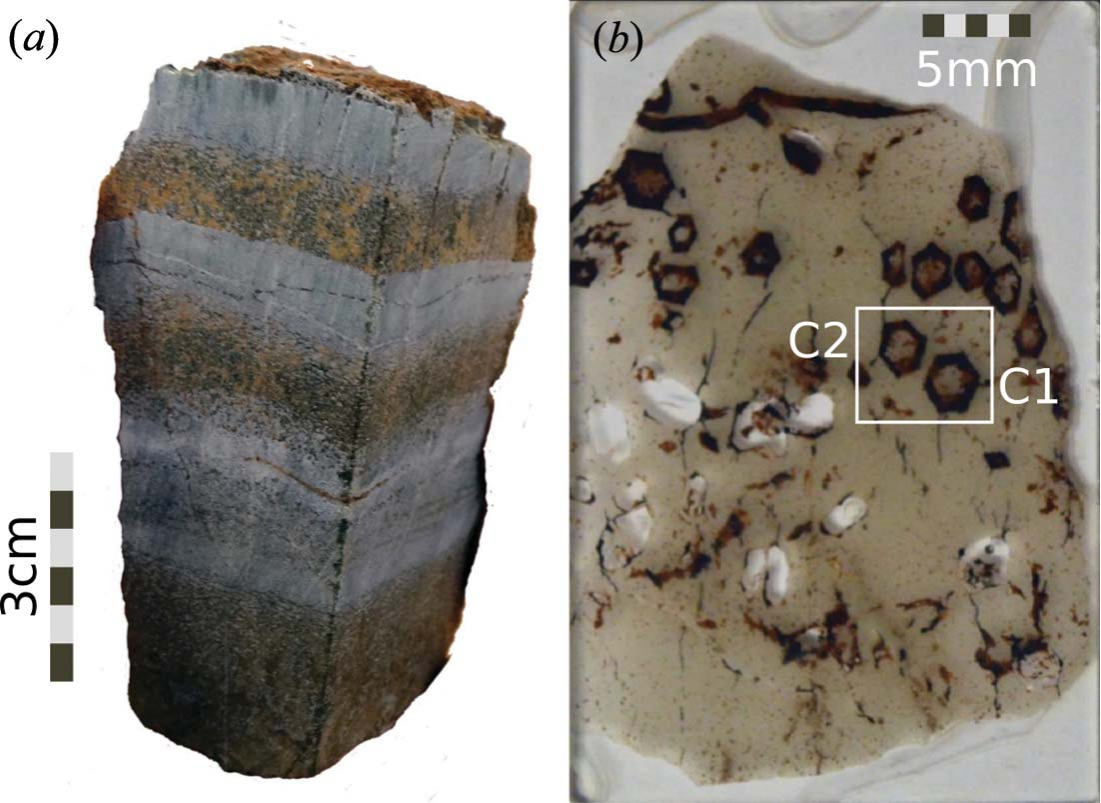
Samples studied in this work. (*a*) ‘Zebra rock’ sample used for the computed tomography study. Crystal pseudomorphs are located in the bottom brown layer. (*b*) Thin section of the rock used for the microscopy and EDX studies. Note the pseudohexagonal outline of the crystals. The white rectangle encloses the two crystals investigated in the EDX study (Fig. 4 ▸).

The rock sample used in the study (Fig. 2 ▸) is from a 5 m thick section of the lower (North Pole) chert member of the Dresser Formation (Van Kranendonk, 2000 ▸), from stratigraphic section C of Van Kranendonk *et al.* (2008 ▸), and belonging to Lithostratigraphic Assemblage 2 of Djokic (2015 ▸). This assemblage is a package of shallow water deposits including the carbonate-chert ‘zebra rock’ near the base, overlain by rippled sandstone and stromatolites.

**Figure 3:**
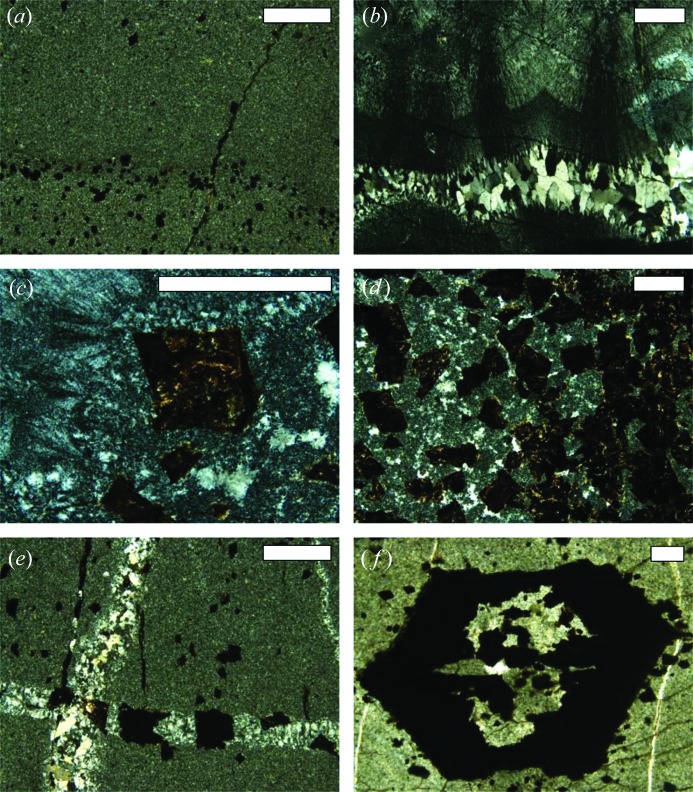
Representative optical photomicrographs from the petrographic study of the ‘zebra rock’ samples. (*a*) Micro-crystalline quartz surrounding the pseudomorphs. (*b*) Layers of coarse quartz and fibrous quartz perpendicular to the surface. (*c*), (*d*) Euhedral carbonate crystal rhombs in the carbonate layers. (*e*) Thin veins of medium to coarse quartz across the microcrystalline quartz layers. (*f*) Large pseudomorphed hexagonal crystal filled with the matrix material; notice the concave stepped faces (especially the top and bottom ones). All scale bars are 0.5 mm in width.

Fig. 2 ▸(*a*) shows the sample selected for microscopic, tomographic and X-ray diffraction studies. This sample was cut to obtain slices suitable for optical microscopy studies and electron microscopy energy dispersive X-ray (EDX) studies (Fig. 2 ▸ *b*). In the outcrop, as well as in sample cross sections, crystals appear to be hexagonal, or pseudohexagonal, and most are hollow. The ‘zebra rock’ hosting the crystals is a fine-grained mixture of microquartz and rhombic carbonate mineral crystals that consist of an unusually Mn-rich carbonate (García-Ruiz *et al.*, 2003 ▸). Textural analysis of the sample (Fig. 3 ▸) shows that the grain size of the matrix surrounding the pseudomorphs (Fig. 3 ▸ *a*) is too small to allow us to identify individual phases, and it will be herein referred to as microcrystalline quartz, although it also contains Al-rich phases, probably micas.

Locally, some concordant layers of coarse columnar quartz occur, with quartz fibrous texture oriented perpendicular to the surface (Fig. 3 ▸ *b*). The transition from the conchoidal quartz growth zone to zones with microquartz and euhedral carbonate pseudomorphs is sharp. Within the bulk of the carbonate layers, zones of equigranular microquartz coexist with patches of coarser-grained fibrous-textured quartz (Fig. 3 ▸ *c*). The transition from carbonate beds to overlying chert beds is gradational, whereas the contact between chert beds and overlying carbonate beds containing carbonate crystals in microquartz is sharp (Fig. 3 ▸ *a*). Zones with abundant euhedral carbonate crystal rhombs lie in a matrix of microcrystalline quartz and patches with coarse-grained quartz (Fig. 3 ▸ *d*).

Thin veins of medium-coarse quartz locally cut across beds of microcrystalline quartz (Fig. 3 ▸ *e*). These veins, which contain large pyrite crystals (pseudomorphed by limonite–goethite), show mosaic or columnar quartz textures.

Pseudomorphs of the large (more than 500 µm) hexagonal crystals forming splays are found within the rock (Figs. 1 ▸ *c*, 2 ▸ *b* and 3 ▸ *f*). They are amorphous and hollow with an opaque (in cross-polarized light) outer rind of Fe-oxy-hydroxides and a core zone of fine-grained (microcrystalline) quartz. The opaque rinds contain neither ores (sulfides or oxides) nor silicates. Chemical analysis of this area (see below) shows that it is rich in Ca, Mg and Fe, including, most likely, amorphous Mg–Fe hydroxides.

**Figure 4:**
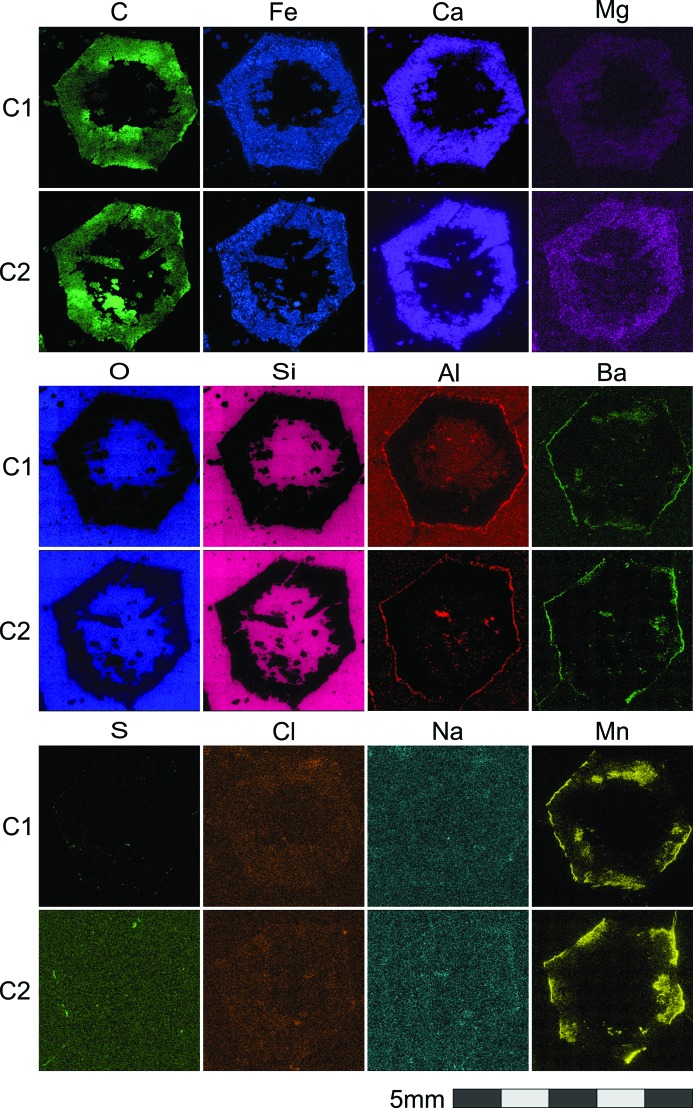
EDX analysis of two pseudomorphed crystals (labeled C1, C2 in Fig. 2 ▸), showing concentration maps collected for C, Fe, Ca, Mg, O, Si, Al, Ba, S, Cl, Na and Mn (from top to bottom and from left to right).

Chemical characterization of the rock slice shown in Fig. 2 ▸(*b*) was performed using EDX spectroscopy. Fig. 4 ▸ shows the compositional maps for C, Fe, Ca, Mg, O, Si, Al, Ba, S, Cl, Na and Mn. *K* series peaks were used in all cases except for Ba, were *L* series were used. These maps show a clear distinction in chemical composition (except for Cl and Na) between the pseudomorphs and the surrounding microcrystalline quartz matrix. The pseudomorphed crystal rim around the hollow core contains C, Fe, Ca and Mg, whereas the surrounding matrix is mostly made of Si, O and Al. Ba is present mostly as a coating of the crystal surface, together with Al and Mn. Mn is also present in the crystal volume, but not homogeneously. Al also shows homogeneously in the matrix in addition to the surface coating. S is present in a few grains at the crystal surface and in discrete regions of the crystal, located at both the outer and the inner surfaces of the hollow pseudomorphs. Cl and Na appear almost homogenously distributed in the crystals and the matrix, being slightly more concentrated in the crystal volume.

**Figure 5:**
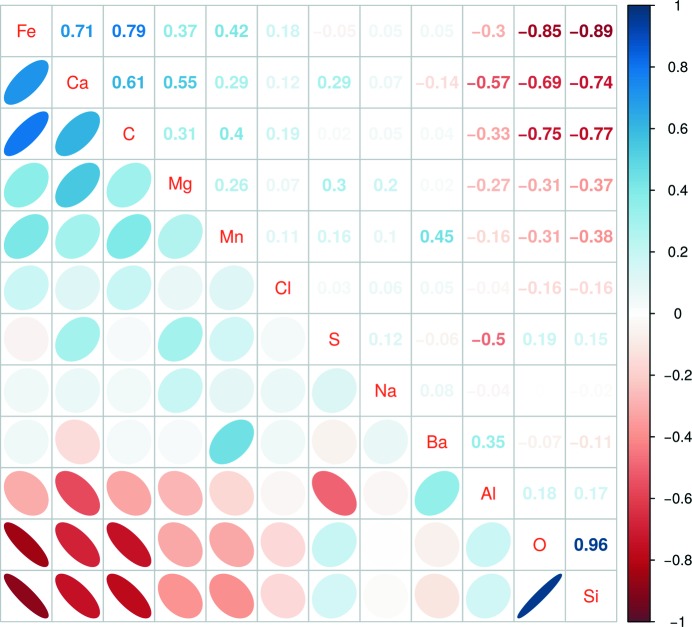
Compositional correlations between the concentrations of different elements obtained by EDX. The upper triangle lists the numerical value of each correlation coefficient; the lower triangle contains a sketched representation of the correlation level and sign. Elements in the diagonal are ordered from top to bottom in descending order of the first principal component so that elements with high positive and negative correlations are located, respectively, at the top and bottom of the table. The color bar to the right shows the color mapped correlation coefficients used in both triangles.

These data are also shown as a correlation plot in Fig. 5 ▸. The strongest correlation is observed between O and Si concentrations, owing to the abundance of quartz in the matrix and in the core of the crystals. Significant to strong positive compositional correlations are also observed between the elements Fe, Ca, C and Mg. Significant to strong negative correlations are observed between Si, O and Al, and the elements of the first group (Fe, Ca, C and Mg). These two groups of elements identify the main components of the pseudomorphed crystals, their outer rind *versus* the matrix and crystal cores, respectively. The concentrations of Mn, Cl, S, Na, and Ba are poorly correlated with those of the other elements, with the exception – as previously noted – of the correlation between Ba and Mn concentrations.

**Figure 6:**
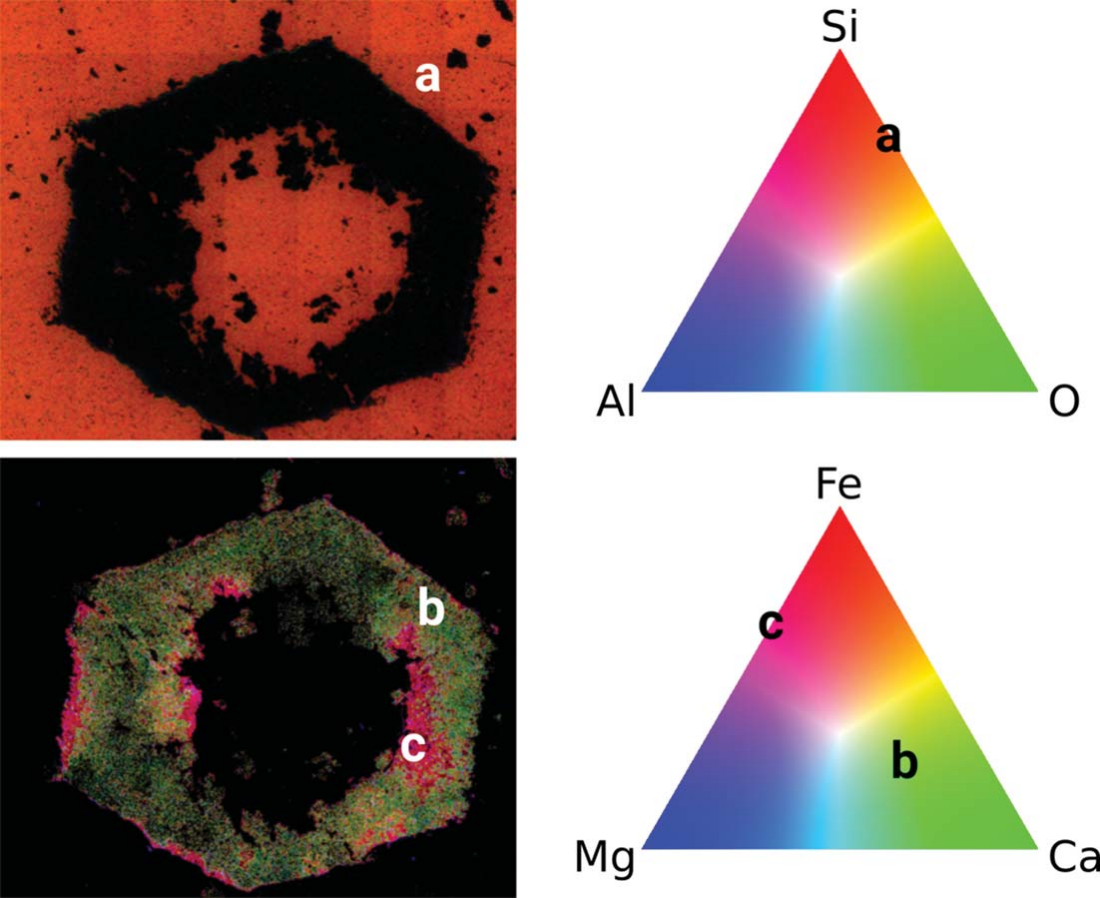
Color maps showing the contribution of different element combinations to the matrix and core of the pseudomorphed crystals (top) and the outer rind of the pseudomorphed crystals (bottom). Each pixel of the matrix map (top) is colored by an RGB combination proportional to the concentrations of Si, Al and O, as illustrated in the top-right triangle. The pixel color in the pseudomorph map (bottom) combines the concentrations of Fe, Ca and Mg in the same way, but additionally, the color intensity (color value) encodes the C concentration to distinguish regions containing carbonates. Two distinct areas within the outer rind are indicated by ‘b’ and ‘c’.

Fig. 6 ▸ summarizes all these observations in terms of ‘characteristic compositions’ of the two different regions. The matrix (composition marked ‘a’ in the plot) is very homogeneous, being composed of quartz with some micrograins of Al-bearing phases. Within the outer rinds of the pseudomorphed crystals, however, two different regions can be clearly distinguished: the largest one (marked ‘b’) composed of Ca–Mg–Fe carbonate (Ca being more abundant), and a second region (marked ‘c’), located in both the outer coating and the inner surfaces of the hollow crystals, where Fe, Mg and Mn are concentrated but Ca is almost absent. These latter regions could be a mix of amorphous or microcrystalline Fe oxides/hydroxides, although C is present too. Ba is also concentrated in these Fe–Mg–Mn-rich regions, as well as the few gains containing S. The consistency of the textural and chemical information shown in this section was checked by X-ray powder diffraction analysis of the mineral phases in the sample. These results are shown in the supporting information.

## 3. Crystal habit   

The crystal morphology and habit of the pseudomorphs from the Dresser ‘zebra rock’ unit was investigated as a proxy to identify the primary mineral. Assigning the primary mineralogy of the Precambrian pseudomorphs by comparing crystal habit is not straightforward (Babel & Schreiber, 2013 ▸). For example, characteristically lens-shaped crystal pseudomorphs interpreted as gypsum could also correspond to other minerals, such as ikaite, gaylussite (Warren, 2006 ▸) or glauberite, which all have similar morphologies to gypsum (Salvany *et al.*, 2007 ▸). Similarly, both trona and gypsum form radial sprays of monoclinic crystals (Smoot & Lowenstein, 1991 ▸). In addition, some groups of minerals can form similar pseudomorphs: ikaite–gypsum–gaylussite–glauberite, barite–siderite–gypsum, aragonite–gypsum, pyrite–halite–sylvite and anhydrite–gypsum (Warren, 2006 ▸; Babel & Schreiber, 2013 ▸). Among them, aragonite, gypsum and nahcolite are especially relevant because crystals of these phases have been reported within Archean deposits and because the ratio between carbonates and evaporites is used to interpret the geochemistry of the original depositional environment (Hardie, 2003 ▸). Thus, the use of crystals as proxies for the chemical conditions of the Archean seawater requires an unambiguous interpretation of their original mineral phase. We have considered a list of plausible candidate minerals suggested by Lowe & Worrell (1999 ▸) and Sugitani *et al.* (2003 ▸). We have not considered for further analysis barium carbonate (witherite) and strontium carbonate (strontianite) – isomorphs with aragonite – because calcium is by far the most abundant alkaline-earth metal in the composition of the psuedomorphs. The crystal forms included in the crystal habit were checked and the theoretical interfacial angles were computed [using the morphological methods described by Dowty (1980 ▸)] from the unit-cell data available in the American Mineralogist Crystal Structure Database (http://rruff.geo.arizona.edu/AMS/amcsd.php). Minerals, unit cells, forms and computed interfacial angles are shown in Table 1 ▸.

**Figure 7:**
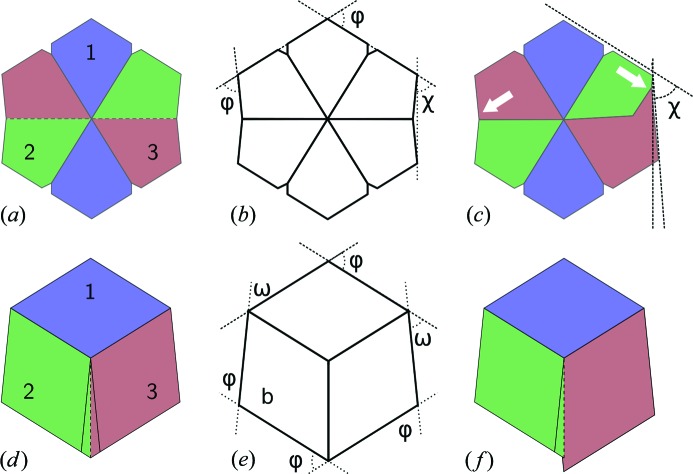
Theoretical morphology of the penetration (*a*) and contact (*d*) twins of aragonite. The three individuals involved are encoded by color; noncrystallographic limits between individuals are shown as dashed lines. The characteristic angles of these pseudohexagonal prisms are shown in (*b*) and (*e*), respectively: φ = 63.824°, χ = 58.088°, ω = 52.352°. Note that penetration twins produce concavities in two opposite faces, and a larger spread of interfacial angle measurements for the pinacoid/prism angle (because the ‘pinacoid’ surface is not a real crystallographic face). In contrast, the contact twin produces protruding spurs (*f*).

Note that aragonite is a special case because it can crystallize in two different pseudohexagonal twins, namely the contact and the penetration (110) twins (Makovicky 2012 ▸; Aquilano *et al.*, 1997 ▸). Fig. 7 ▸ shows the cross section of penetration (*a*) and contact (*d*) twins and the corresponding interfacial angles measured in this study (Figs. 7 ▸ *b* and 7 ▸ *e*, respectively). In both cases, these twins produce pseudohexagonal prisms made of three individuals twinned pairwise (1 and 2, 1 and 3, but not 2 and 3). The main difference between the two twins is the distribution of angles: the penetration twin section contains two 63.824° (φ) and four 58.088° (χ) angles, while the contact twin contains four 63.824° (φ) and two 52.352° (ω) angles. The lack of twinning between individuals 2 and 3 produces protruding spurs in the contact twin (Fig. 7 ▸ *f*), while in the penetration twin the lack of twinning between 2 and 3 produces concavities in two opposite faces, as well as a larger spread of interfacial angle measurements for the pinacoid/prism angle (Fig. 7 ▸ *c*).

To reconstruct the habit of the primary crystals, the Dresser sample was analyzed by X-ray microtomography, using an EasyTom XL Duo instrument developed by the company RX Solutions. A sealed Hamamatsu microfocus X-ray source was used, coupled to a Varian PaxScan 2520DX detector (flat panel with amorphous silicon and a CsI conversion screen; 1920 × 1536 pixel matrix; pixel pitch of 127 µm; 16 bits of dynamic range). The entire sample was scanned in a vertical stack mode (4320 projections in three turns) with a spatial resolution of 39.98 µm. Parameters of the acquisition were 50 kV (tube voltage), 500 µA (tube current), ten frames per second, averaging of 20 frames per projection, filtration of the beam by 0.3 mm of copper foil, a source-to-detector distance of 372 mm and a source-to-object distance of 117 mm. From these data we tried three different methods to measure angles from (*a*) the three-dimensional segmented models provided by the tomographic software, (*b*) a fit of a set of segments defined on slices of the raw data and (*c*) crystal outlines defined from the projection of oriented volumes containing the crystals. Methods (*a*) and (*b*) produced angular data with a large spread and are presented in the supplementary methods for their didactic relevance. Method (*c*) produced a data set with a smaller spread that has been used for the rest of the paper.

**Figure 8:**
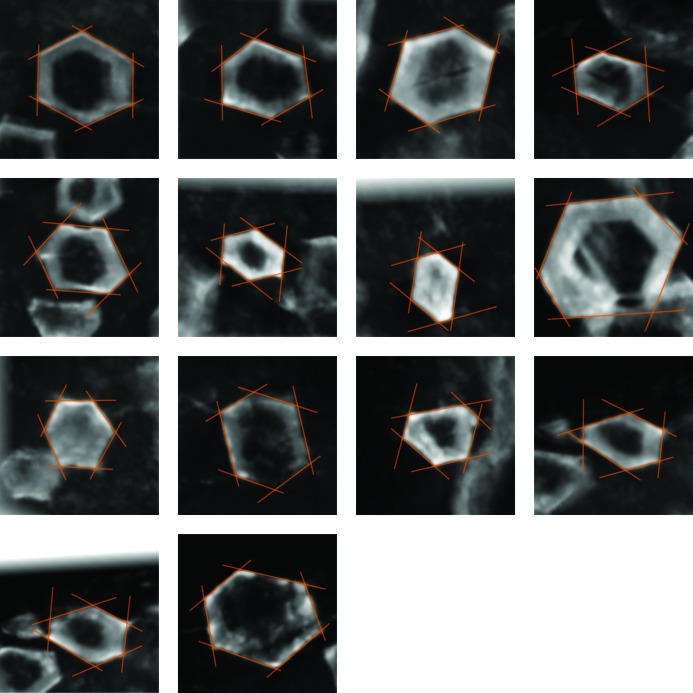
The 14 crystal projections used to measure accurate interfacial angles. These pictures are projections through the three-dimensional stack of slices (between 15 and 50 slices in each case) after rotation of the voxel array. The rotation angles were computed from the orientation matrix and then visually optimized to maximize the contrast of the crystal sections, which corresponds to perfect alignment of the prism zone axis parallel to the view direction. The process is similar to setting the crystal ‘in focus’, as can be seen by comparing the aligned crystals with other crystals included in the field of view but not oriented. The morphology of the aligned sections (orange lines) was defined and used to compute the interfacial angles.

Interfacial angles were measured from a set of 14 crystals (84 angles in total) after rotating and projecting the voxel volume obtained from tomography so that the elongation of the crystal was perpendicular to the image. The rotation angles used for this orientation were obtained by minimizing the projected convex hull of the set of segments obtained from the analysis of crystal outlines in tomographic sections [method (*b*), see supplementary materials] and later visually optimized within ±0.5° in a process similar to getting the image in focus (Fig. 8 ▸). To guarantee the accuracy of crystal orientation and angular measurements, a minimum number of slices was required so that the stack of slices spans a height larger than the crystal diameter.

**Figure 9:**
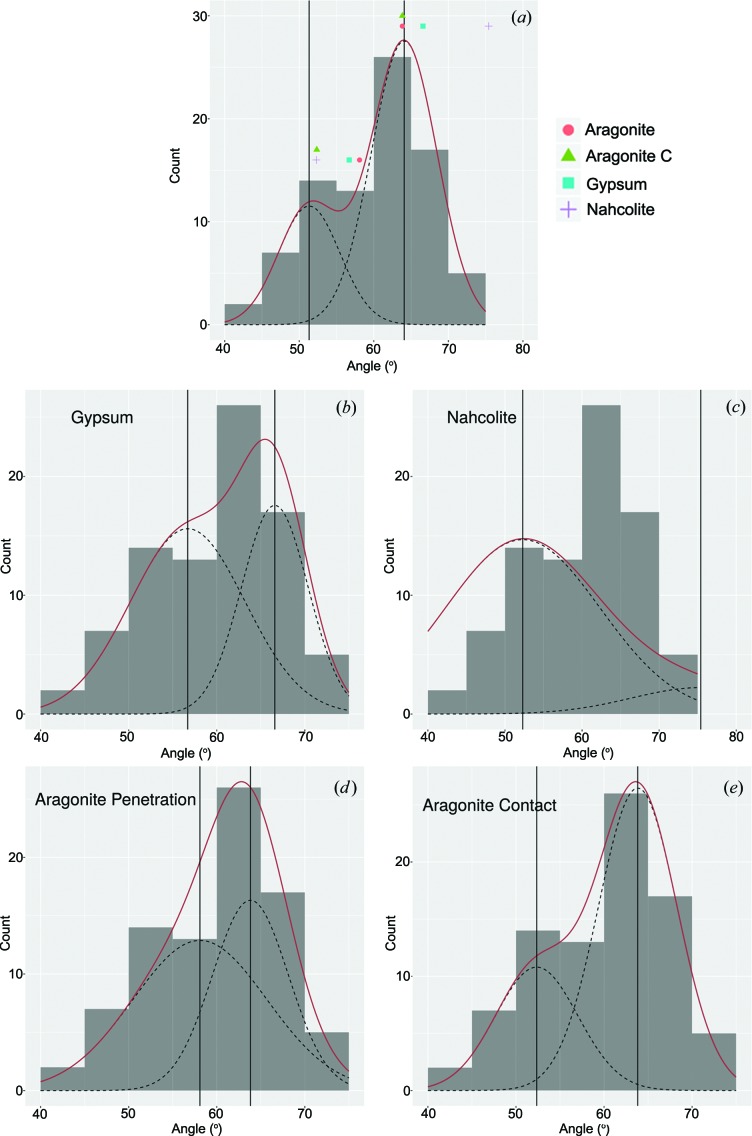
The bimodal histogram of the experimentally measured angles (*a*) showing two main maxima, and their fitting to the main mineral candidates (*b*)–(*e*). The dashed lines are normal distributions centered on the two theoretical angles for each mineral. The red line is the summation of both distributions. The vertical solid lines mark the maximum of each of the distributions at the fixed values for each mineral. In (*a*) the solid black lines mark the maxima of the two normal distributions, and the position of the calculated interfacial angles for gypsum, aragonite and nahcolite is also shown.

Fig. 8 ▸ shows the 14 rotated and projected volumes used for the analysis, along with the faces defined for each of the crystals. All interfacial angles were measured, and the distribution of these angles is shown in Fig. 9 ▸. The angle distribution is clearly bi-modal, with maxima of probability at 51.331 and 64.076° (Fig. 9 ▸ *a*) obtained by the unbiased expectation-maximization (EM) algorithm (Benaglia *et al.*, 2009 ▸). This means that, despite the apparent hexagonal morphology of the crystals, they are more properly interpreted as pseudohexagonal crystals or twins. Fig. 9 ▸(*a*) also shows the theoretical angles expected for the three minerals that are most likely to constitute the original phase during the growth of the crystals: nahcolite, gypsum and the two twins of aragonite.

For the fitting in Fig. 9 ▸(*a*), the EM algorithm was set to fit five parameters, namely the position of the maxima of the normal distributions μ_1_, μ_2_, the spread (standard deviation) of the distributions σ_1_, σ_2_, and the relative weight of the two distributions λ_1_, (1 − λ_1_). The total distribution obtained from the fitting (solid red line in Fig. 8 ▸) is then *P* = λ_1_ *N*(μ_1_, σ_1_) + (1 − λ_1_)*N*(μ_2_, σ_2_), where *N*(μ, σ) is the normal distribution with mean μ and standard deviation σ. The prism/prism (110^1
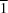
0) angle of the penetration and contact twins of aragonite fits the maximum of the distribution very well. The corresponding pinacoid/prism angle is relatively far from the second maximum at lower angles in the case of the penetration twin but very close in the case of the contact twin. The fitting for nahcolite is very good for the pinacoid/prism angle but very bad for the prism/prism angle. Finally, gypsum falls in between; neither of the two angles is at the maximum of the distribution, but both are reasonably close. In order to quantitatively assess the likelihood of each individual mineral, we performed fits to a mixture of two normal distributions having a fixed position at the theoretical angles computed for each of them, *i.e.* μ_1_ and μ_2_ were fixed to the theoretical values of each of the minerals and the other three parameters were fitted. Fig. 9 ▸(*b*)–9 ▸(*d*) show these fits.

The two twins of aragonite show a maximum corresponding to the measured angles and a good fit to the overall asymmetric distribution. The distribution obtained by fitting the gypsum angles is also reasonable but worse; the overall shape is reproduced, but the position of the maximum is displaced. The fitting to the angles of nahcolite is very bad, mainly because the theoretical value of the prism/prism angle is out of the range of the measured angles. The goodness of these fits has been quantified by computing p-values using the one-sample Kolmogorov–Smirnov test (Conover, 1971 ▸). The output of this significance test is shown in Table 2 ▸.

Data from Table 2 ▸ show that the primary crystals were probably aragonite (p-value = 0.66 for penetration twin and 0.69 for contact twin). Gypsum (p-value = 0.44) has a much lower probability, and nahcolite can be fully discounted. The fact that the highest p-value is found for the twins of aragonite is also reflected in the standard deviation values. Pseudohexagonal aragonite prisms are cyclic twins containing three individuals twinned in pairs (Fig. 7 ▸). Individual 1 is twinned with 2 and 3, but 2 and 3 are not twinned with each other; therefore, the vertical faces in Fig. 7 ▸(*c*) are not crystallographic faces but are made of segments of subparallel faces from two non-twinned individuals meeting in this face. This produces lattice strain and concavities in the crystal faces that are observed in some of the cross sections [see for instance Figs. 3 ▸(*f*) and 10 ▸(*b*)] and that contribute to the intrinsic roughness of the surface of pseudomorphs. Fig. 9 ▸ and Table 2 ▸ show that, in both gypsum and the penetration twin of aragonite, the standard deviation of the distribution fitting the pinacoid/prism angle is almost twice the standard deviation of the distribution fitting the prism/prism angle. There is no reason for observing these differences in a set of interfacial angle measurements between crystallographic planes because the angles in crystals are constant and well defined. The standard deviation of the distribution can only be attributed to the characteristics of the sample or the intrinsic experimental errors, which should be equal for both distributions. A reasonable explanation for this difference is, again, that the crystals were aragonite originally. Fig. 7 ▸(*a*) shows a cross section of the aragonite penetration twin with the three individuals of the twin growing at exactly the same rate. This is a very unlikely ideal situation. Often, one of the individuals will grow slightly faster than the other, and therefore the morphology will develop as illustrated in Fig. 7 ▸(*c*). The surface where the non-twinned individuals 2 and 3 meet (right side) is composed mainly of individual 2 and, as a consequence, the measured angle between the pinacoid and the prism face will decrease by up to 2.2° depending on the relative development of each individual. Differences in growth rate, leading to differences in the relative development of the faces of individuals 2 and 3, are expected and could be the reason for the larger standard deviation in the observed pinacoid/prism interfacial angle distribution. When considering the contact twin a better agreement is obtained (Fig. 9 ▸ *e* and Table 2 ▸). The maxima of the measured angle distribution are very close to the theoretical angles, the standard deviation of both normal distributions is very close, the relative weight (λ) of both distributions is close to the expected ratio (1/3 of the measurements close to the 52.352° angle, 2/3 close to 63.824°) and the p-value is the highest of all models (0.694). Therefore, we conclude that the measured interfacial angles come from a combined population of pseudohexagonal aragonite penetration and contact twins.

**Figure 10:**
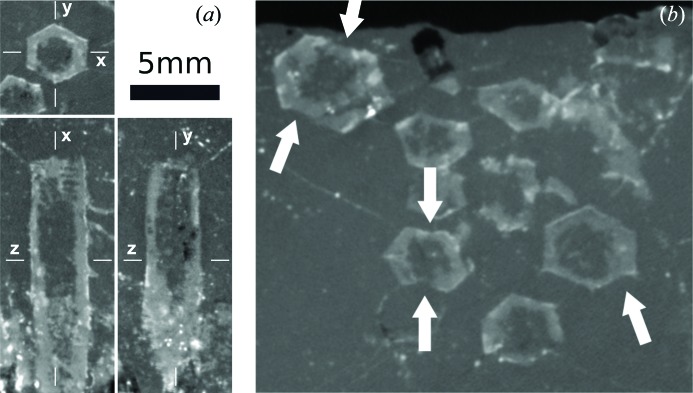
(*a*) Three orthogonal views of the crystals to show the crystal terminations out of the prism zone. (*b*) A detail of the CT images highlighting two typical features of the pseudomorphs: (*a*) the concavities in some faces of the prisms, which are characteristic of the penetration twins, and (*b*) pointed spurs close to some vertices, which are a typical feature of aragonite contact twins (see also Fig. 7 ▸).

There is also another observation in favor of aragonite *versus* other candidates, which is related to the termination of the pseudohexagonal prism. Aragonite twinned prisms most commonly end with (001) faces perpendicular to the prism axis, while gypsum crystals normally end with (011) faces, making a step angle with the zone axis. These (011) faces can also form a marked reentrant angle if the gypsum crystal is twinned. Some terminations of the pseudomorphs can be observed from the volumetric data (Fig. 10 ▸ *a*), showing that they are made of single surfaces perpendicular to all prism faces, close to what is expected from aragonite and far from the typical terminations of gypsum crystals.

## 4. Conclusions   

We show that the analysis of computed X-ray tomography images of rocks containing single crystals can be used to identify mineral phases by a morphological crystallographic analysis. The measurements to be used in the morphological study need to be obtained from a detailed analysis of the raw three-dimensional absorption data because the measurements yielded by the automatic processing by tomography software – defined by default to create images – are not accurate enough for crystallographic analysis.

When this method was applied to the pseudomorphed radiating crystal splays in the interbedded carbonate and chert ‘zebra rock’ unit of the lower Dresser Formation, we found that they were originally hollow aragonite pseudohexagonal twins (Figs. 9 ▸ and 10 ▸). Gypsum is also a possible candidate that cannot be completely excluded, but the statistical analysis of interfacial angle distribution and all the additional crystallographic and chemical results point to aragonite. Nahcolite can be excluded conclusively as a candidate mineral on the basis of interfacial angle measurements. In summary, we conclude that the measured angle distribution is explained by the interfacial angles of contact and penetration twins of aragonite better than any other of the minerals considered. Other crystallographic features like the crystal termination faces out of the [001] zone and the presence of concave faces support this conclusion. The morphology of the pseudohexagonal aragonite prisms also explains the width of the measured interfacial angle distribution. Furthermore, the hollow morphology of the prisms is consistent with the higher strain in the aragonite crystal lattice due to imperfect twinning. Actually, hollow aragonite prismatic twins have been reported in current lacustrine evaporitic environments (Jones & Renaut, 1996 ▸) but have never been reported for sedimentary or evaporitic gypsum. Chemical mappings by EDX also support the conclusion obtained from crystal morphology.

Our results show that it is possible to identify, using morphological crystallographic analysis, the original mineral phases of fully silicified pseudomorphs of Archean evaporitic crystals. The method used in this work could be applied to many other crystal patterns reported within Precambrian rocks. The identification of the original mineral phases of Archean crystal patterns will contribute precious information about the geochemical constraints at the onset of life on this planet.

## Supplementary Material

Supporting information. DOI: 10.1107/S1600576718007343/jo5036sup1.pdf

Video S1 shows a stream of virtual cross sections obtained by X-ray microtomography. DOI: 10.1107/S1600576718007343/jo5036sup2.mp4

## Figures and Tables

**Table 1:** Crystallographic data of the candidate pseudo-hexagonal minerals The columns contain the crystal system S (O = orthorhombic, M = monoclinic), the unit-cell parameters (*a*, *b*, *c*, β), the pinacoid face entering the pseudo-hexagonal morphology and the corresponding prism faces. The last two columns contain, respectively, the pinacoid/prism angle and the prism/prism angle. For aragonite, the (110) penetration and contact twins have been used for calculations. In the penetration twin, the ‘pinacoid face’ is made of two slightly misoriented (110) faces having the average orientation of the (010) pinacoid. In the contact twin, the faces labeled ‘pinacoid’ are the (110) faces of the next individual of the twin (*). See Fig. 7 ▸ for further discussion

Mineral	S	*a*	*b*	*c*	β	pin	pri1	pri2	pin^pri	pri1^pri2
Barite	O	8.8842	5.4559	7.1569	90	(001)	(011)	(01 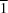 )	52.6807	74.6385
Celestine	O	8.3770	5.3500	6.8730	90	(001)	(011)	(01 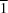 )	52.1025	75.7950
Gypsum	M	6.2840	15.2000	6.5230	127.410	(010)	(120)	(1 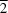 0)	56.7044	66.5912
Mirabilite	M	1.5120	10.3700	12.8470	107.789	(010)	(110)	( 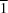 10)	43.4114	86.8228
Aragonite (penetration)	O	4.9614	7.9671	5.7404	90	(010)	(110)	(1 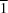 0)	58.0880	63.8240
Aragonite (contact)	O	4.9614	7.9671	5.7404	90	(110)*	(110)	(1 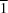 0)	52.352	63.8240
Strontianite	O	5.1075	8.4138	6.0269	90	(010)	( 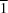 10)	( 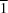 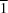 0)	58.7407	62.5186
Nahcolite	M	7.5100	9.7000	3.5300	93.320	(010)	(110)	(1 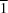 0)	52.2986	75.4028
Borax	M	1.8580	10.6740	12.1970	106.680	(010)	( 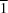 10)	( 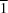 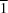 0)	43.2191	93.5617

**Table 2:** Statistics from the fits shown in Fig. 9 ▸ The columns correspond to Figs. 9 ▸(*a*), 9 ▸(*b*), 9 ▸(*d*), 9 ▸(*e*) and 9 ▸(*c*) from left to right. The rows show the relative amplitude (λ), position (μ) and standard deviation (σ) of the two distributions fitted to the experimental data [free μ in Fig. 9(*a*)] and the three minerals [fixed μ in Figs. 9(*b*)–9 ▸(*e*)]. The last two rows show the Kolmogorov–Smirnov statistic (*D*_n_) and the asymptotic significance (p-value) of the test. The *D*_n_ value and p-value in the first (‘Data’) column correspond to the KS test of the free fitting shown in Fig. 9 ▸(*a*) and are therefore the baseline for the other values.

	Data	Gypsum	Aragonite (penetration)	Aragonite (contact)	Nahcolite
λ(pin^pri)	0.28335	0.60673	0.58452	0.30120	0.88216
μ(pin^pri)	51.3306	56.7044	58.0880	52.3520	52.2986
σ(pin^pri)	4.12066	6.51756	7.60615	4.67518	10.0672
λ(pri^pri)	0.71665	0.39327	0.41547	0.69880	0.11784
μ(pri^pri)	64.0756	66.5912	63.8240	63.8240	75.4028
σ(pri^pri)	4.41147	3.79683	4.31637	4.47695	8.97991
*D*_n_	0.06257	0.09482	0.079616	0.07750	0.35599
p-value	0.8973	0.4369	0.6614	0.69410	1.13 ×10^−9^
