# Supplementary material for: A crystallographic study of crystalline casts and pseudomorphs from the 3.5 Ga Dresser Formation, Pilbara Craton (Australia)
Source: J Appl Crystallogr. 2018 Jul 5;51(Pt 4):1050–8. doi: 10.1107/S1600576718007343 (PMC6068616; doi:10.1107/S1600576718007343)
Supplement: Supplementary file 1 [file j-51-01050-sup1.pdf]

JOURNAL OF APPLIED CRYSTALLOGRAPHY

Volume 51 (2018)

Supporting information for article:

A crystallographic study of crystalline casts and pseudomorphs from the 3.5 Ga Dresser Formation, Pilbara Craton (Australia)

Fermin Otálora, A. Mazurier, J. M. Garcia-Ruiz, M. J. Van Kranendonk, E. Kotopoulou, A. El Albani and C. J. Garrido

Supplementary Material

Powder X-Ray diffraction analysis

Table 1 and Fig. S1 show the results of the powder X-ray diffraction analysis of the sample. A small volume (5×5×2mm) containing both pseudomorphs and matrix was analyzed, collecting powder diffraction data, identifying the phases present and refining (Rietveld) the full profile. The Fundamental Parameters method was used to simulate the experimental widening function and to extract quantitative microstructural information. Ankerite was identified as one of the end member of a solid solution; the experimental data can also be fitted using the isostructural ferroan dolomite (PDF 34-0517) with the same results. Data fitting is remarkably good, which excludes the presence of any other mineral in significant amounts, and makes quite reliable the quantitative information on the amount of each mineral and the estimation of the grain size. Both ankerite and goethite show as nanoparticles of 20-30nm, as determined from the Rietveld refinement of the peak's widening. The particle size for quartz and calcite (marked with a trailing asterisk in table 1) are not meaningful as they are in practice infinite for x-ray diffraction.

Table 1: Crystalline phases identified in the sample.

**Mineral**	**Quartz**	**Calcite**	**Goethite**	**Ankerite**
**Content**	50.99%	32.74%	8.50%	7.77%
**database code**	COD 9009666	COD 4502443	COD 9011412	PDF 41-0586
**Formula**	SiO_2	$CaCO_3$	FeO_2	$Ca(Fe^{+2},Mg)(CO_3)_2$
**Reference**	(Gualtieri, 2000)	(Zolotoyabko et al., 2010)	(Hoppe, 1940)	(Reeder & Dollase, 1989)
**space group**	P3 2 21	-R 3 2”c	Pbnm	R-3
**unit cell (a,b,c Å)**	4.9158, 4.9158, 5.4091	4.97577, 4.97577, 16.99207	4.59, 10, 3.03	4.8287, 4.8287, 16.1520
**(α,β,γ)**	90, 90, 120	90, 90, 120	90, 90, 90	90, 90, 120
**volume (Å^3)**	113.199	364.332	139.007	326.15
**domain size LVol-FWHM (nm)**	860(30)*	2000(3000)*	33(2)	20(140)

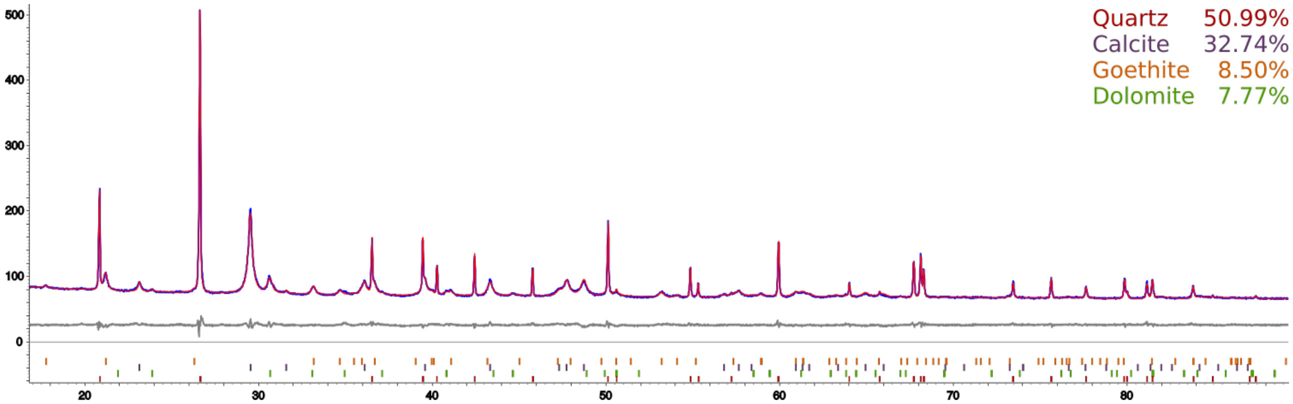

Figure S1. Diffractogram of the sample (pseudomorphs plus matrix around them). Blue line: diffractogram: difractometer Bruker D8 Advance Series II using monochromatic Cu K α1 radiation with a primary Ge (111) monochromator and a PSD Lynxeye detector. 16 hours measurement from 4 o to 90º, 2θ, 0.014 o per second. Peak position of the minerals identified by the EVA software, (labels at top/right) are displayed as small segments at bottom. The theoretical profile, fitted by Rietveld refinement (using Topas 5.0), is shown as a red line that overlaps very closely the experimental results (blue). The differences between the experimental and the computed profiles are shown as a grey line.

Angle measurement from 3D segmented models

Reconstruction of the data has been done with XAct software (RX-solutions) with a classical filtered back projection algorithm and correction of the beam-hardening artifact (Kak & Slanney, 2001). A representative cross-section of this reconstructed volume is shown in Figure S2. Video S1 shows a stream of the computed cross-sections. From these sections, a segmentation was computed, trying to define a surface separating the crystals from the matrix. Due to the presence of veins and clusters of mineral grains in the matrix next to the crystal surface showing the same contrast as the pseudomorphs, a simple automatic segmentation by boundary thresholding was not possible. Instead, a semi-automatic procedure was followed according to the almost regular shape of the crystals. A windowed, non-local means filter (Buades et al., 2005) was first applied in 3D, in order to denoise the data. Then, individually for each of the crystals in the sample, a selection has been done manually on one slice every twenty, before running an interpolation computation between areas selected. The result of this process is presented in Figure S3.

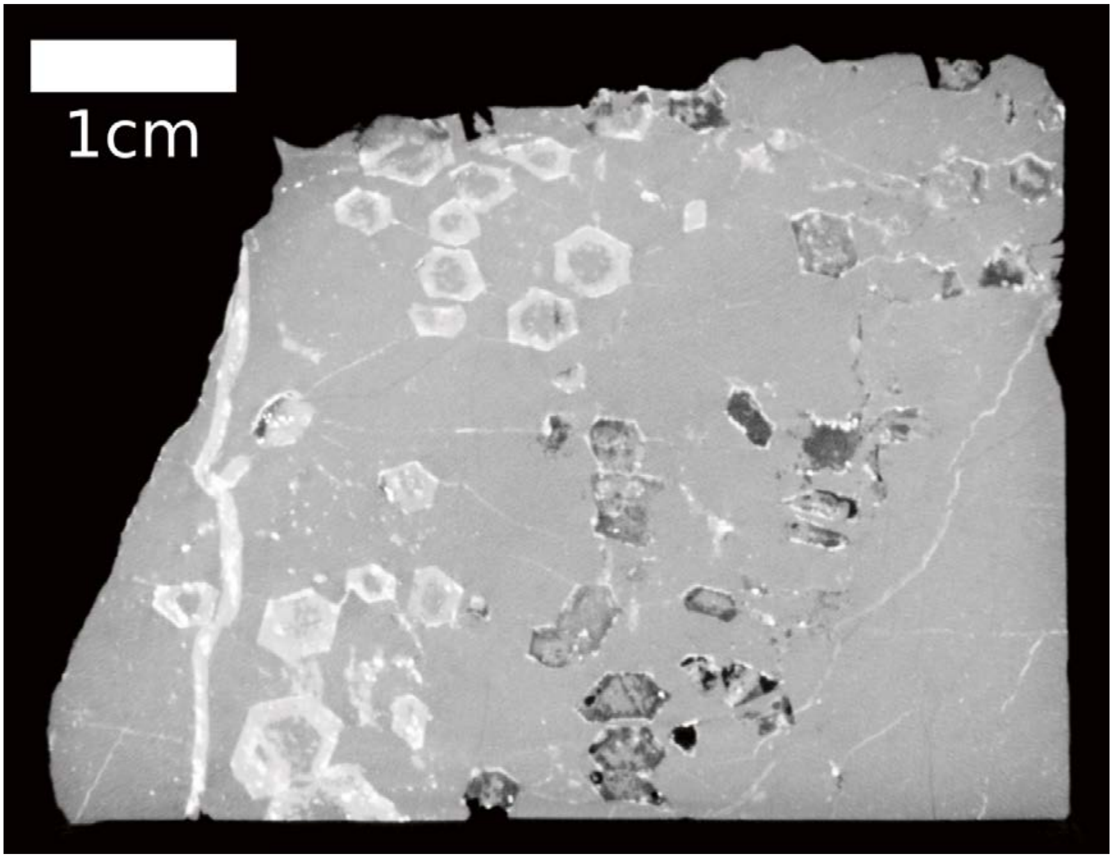

Figure S2. X-ray microtomographic cross-section of the sample shown in Fig. 2a. The picture shows the pseudomorphs, the homogeneous matrix, and some quartz veins of different thickness. Note two visually different types of pseudomorphs. Those fully embedded in the rock shows a brighter colour than the silica matrix outside and inside the hollow crystals. Those crystals exposed to the surface of the sample were weathered), they are now empty and therefore show darker.

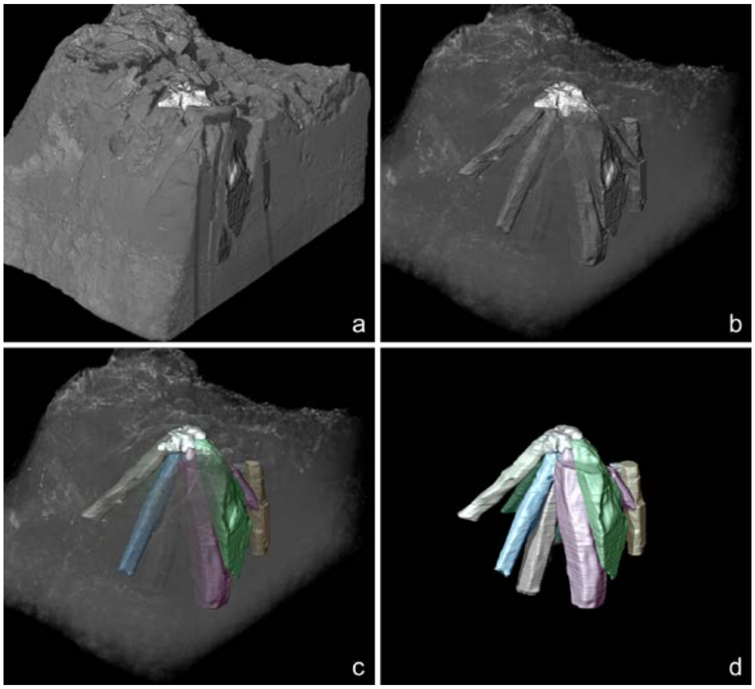

Figure S3. Three-dimensional reconstruction made from the microtomographic volume. The raw 3D data (a) is segmented to extract the volume assigned to the pseudomorphs (b). Each of the individual crystal pseudomorphs in the rosette is identified (c), and a 3D model showing only the crystal surface is finally defined (d).

The crystal surfaces computed from this gray-level segmentation by thresholding the voxel volume were not suitable to measure accurate interfacial angles, due to the apparent intrinsic roughness of the original crystal morphology and the smoothing introduced by the processing described in the previous paragraph. This is probably due to partial dissolution close to the end of the crystal growth process and to additional roughness produced by dissolution or replacement processes during diagenesis and/or metamorphism. Video S1 shows a stream of virtual cross-sections obtained by X-ray microtomography Overall, the reconstructed 3D surfaces were too rough to characterize the crystal morphology by direct measurement of interfacial angles. Crystals were identified as close-to-hexagonal prisms, but the scattering of measurements made the discrimination of hexagonal or pseudo-hexagonal symmetry impossible.

Angle measurement from segments defined in tomography slices

Segments on the crystal outline, as observed in selected slices from the computed tomography reconstructed volume were identified to define shape of each of the 67 crystals identified in the sample in 55 slices spanning the sample volume containing pseudomorphs. These segments are shown in figure S4 .

The dataset containing xyz values of the end points of each segment from each individual face were studied using a principal components analysis by a singular value decomposition of the data, after setting the origin at the mean point of each distribution (crystal face). The obtained matrix of variable loadings (i.e., a matrix whose columns contain the eigenvectors) was used to compute the interfacial angle as the angle between the mid eigenvectors of adjacent faces and the orientation matrix of each of the crystals. The rotation angles were obtained by minimizing the projected convex hull of the set of points obtained from the previous analysis and later visually optimized within ±0.5º in a process similar to getting the image in-focus (Fig. 8). Figure S5 shows a selection of 24 of the crystals after transforming the segments (using the computed orientation matrix) so that the elongation of the crystal and the interfacial edges are perpendicular to the image plane. Unconstrained, non-linear fitting of the points cloud to a set of 3D planes was also tested, computing the interfacial angles from the plane normal vectors. Both procedures produce similar results, with a better statistical significance (smaller spread of the distribution) than the direct measurement from the 3D segmentation. However, interfacial angle values were still too scattered to confidently define the symmetry of the crystals.

The reason for this large data spread was identified to be the existence of a few segments in each dataset that significantly deviate from the mean orientation of the face (see figure S5). These deviations were checked to be due to the interference of mineral grains and veins obscuring the crystal face outline (see figure S2). Instead of just removing the offending segments, we decided to find parts of the prisms completely free of these artifacts.

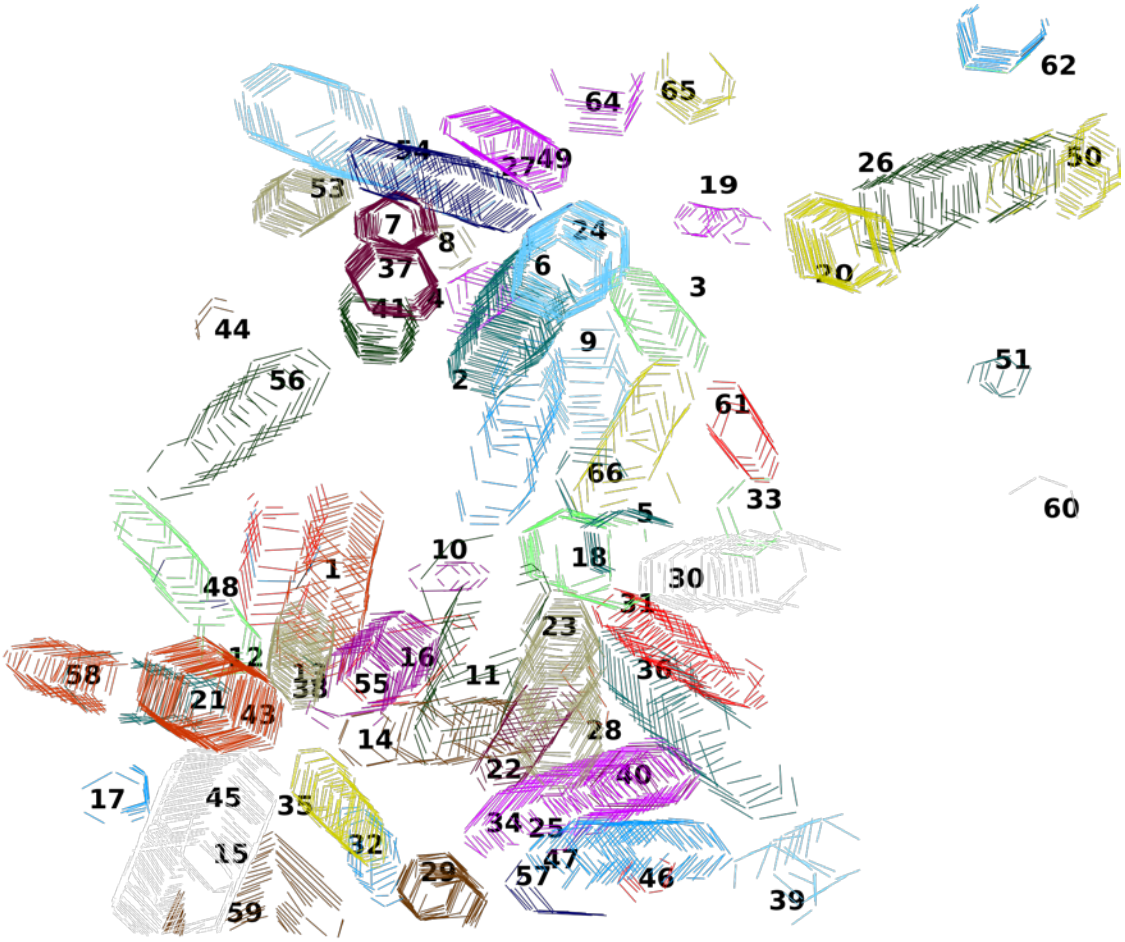

Figure S4. Morphology of the 67 pseudomorphs contained in the sample identified on sections of the computed tomography (unfiltered data). Each segment was identified by crystal number (shown) and section depth.

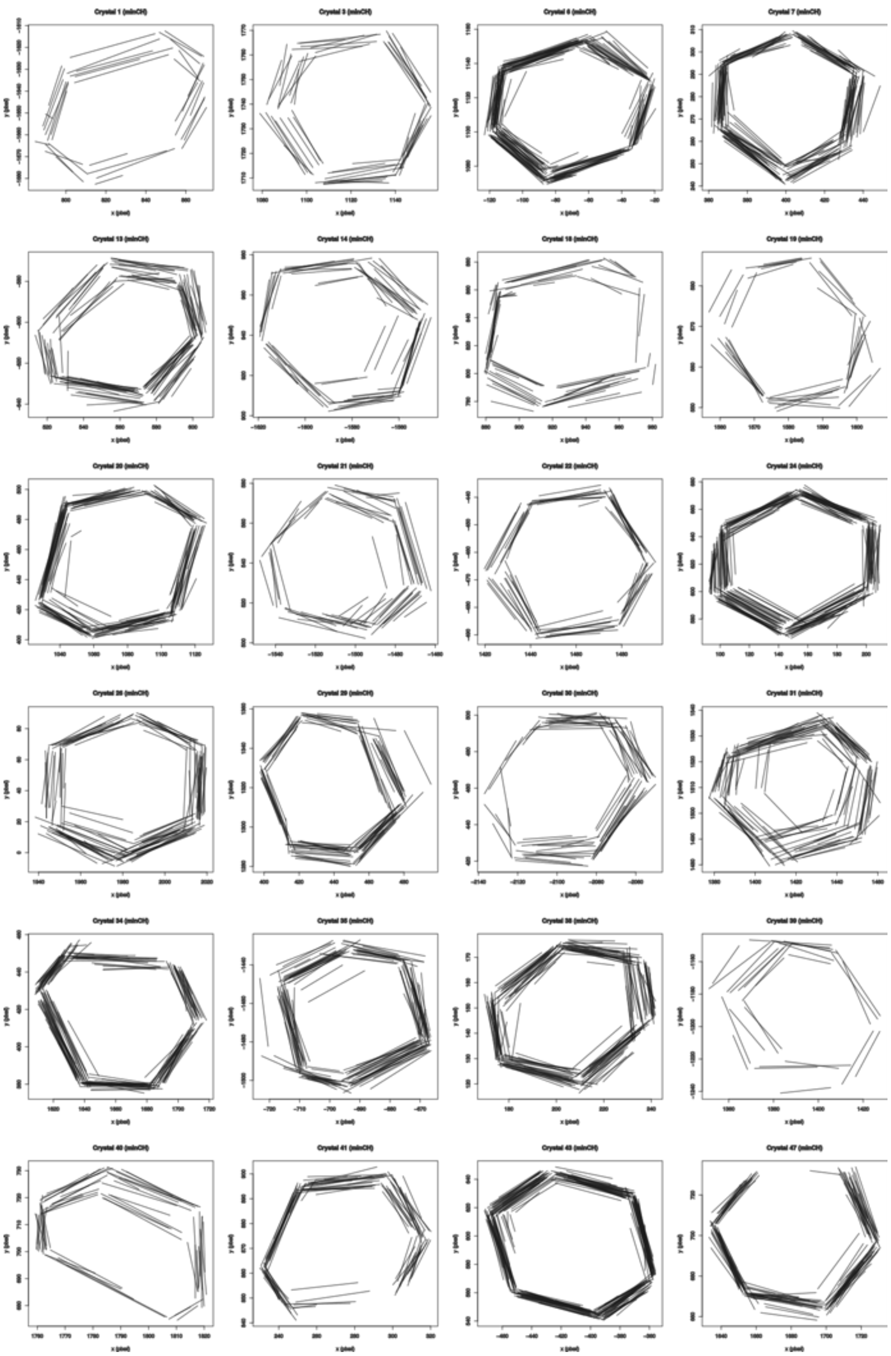

Figure S5. A sample of 24 of the 67 crystals rotated to be perpendicular to the image plane. The orientation matrix for each crystal was computed from data in figure S4.

Table 2: List of the 84 interfacial angle measurements used in the study. Each row corresponds to a different crystal. Column order is meaningless.

62.5	61.0	63.0	54.0	64.5	60.5
58.5	53.0	72.5	46.5	67.5	62.0
47.0	59.5	70.0	52.5	59.5	70.0
43.0	69.0	62.0	54.0	62.0	69.5
52.5	67.5	61.0	48.5	71.0	59.5
51.5	71.0	56.5	52.5	69.5	59.5
54.5	65.0	60.0	55.0	66.5	60.0
52.0	61.5	52.5	63.5	64.0	65.5
63.0	49.5	63.5	64.0	64.0	55.0
49.5	73.0	56.0	56.5	66.0	59.0
53.5	65.0	63.0	55.0	61.5	62.0
44.0	72.5	57-5	49.0	68.0	69.5
45.5	67.0	64.5	53.5	60.0	68.5
52.5	60.0	60.0	60.5	69.0	58.0

References

Buades, A., Coll, B., and Morel, J.M. (2005). In Proceedings / CVPR, pp. 60–65. IEEE Computer Vision and Pattern Recognition 2.

Kak, A. & Slanney, M. (2001). Principes of Computed Tomography Imaging. Society for Industrial and Applied Mathematics. The Institute of Electrical and Electronics Engineers, Inc., New York
